# The Four and a Half LIM-Domain 2 Controls Early Cardiac Cell Commitment and Expansion Via Regulating β-Catenin-Dependent Transcription

**DOI:** 10.1002/stem.1332

**Published:** 2013-01-22

**Authors:** Anke Renger, Maria-Patapia Zafiriou, Claudia Noack, Elena Pavlova, Alexander Becker, Krasimira Sharkova, Martin W Bergmann, Ali El-Armouche, Wolfram-Hubertus Zimmermann, Laura C Zelarayán

**Affiliations:** a Department of Pharmacology and, Heart Research Center Göttingen (HRCG), University Medical Center-Georg-August-University-GöttingenGöttingen, Germany; b Department of Clinical Cardiology, Experimental and Clinical Research Center, Charité Medical Faculty and Max-Delbrück-Centrum for Molecular MedicineBerlin-Buch, Germany; c Department of Cardiology and Pneumology, Heart Research Center Göttingen (HRCG), University Medical Center-Georg-August-University-GöttingenGöttingen, Germany; d Riley Heart Research Center, Herman B Wells Center for Pediatric Research and Krannert Institute of Cardiology, Indiana University School of MedicineIndianapolis, Indiana, USA; e Department of Cardiology, Asklepios KlinikSt. Georg, Hamburg, Germany; f German Center for Cardiovascular Research (DZHK)Partner Site, Göttingen, Germany

**Keywords:** Four and a half LIM-domain 2, β-Catenin, Igfbp5, Mouse embryonic stem cell, Cardiogenesis

## Abstract

The multiphasic regulation of the Wnt/β-catenin canonical pathway is essential for cardiogenesis in vivo and in vitro. To achieve tight regulation of the Wnt/β-catenin signaling, tissue- and cell-specific coactivators and repressors need to be recruited. The identification of such factors may help to elucidate mechanisms leading to enhanced cardiac differentiation efficiency in vitro as well as promote regeneration in vivo. Using a yeast-two-hybrid screen, we identified four-and-a-half-LIM-domain 2 (FHL2) as a cardiac-specific β-catenin interaction partner and activator of Wnt/β-catenin-dependent transcription. We analyzed the role of this interaction for early cardiogenesis in an in vitro model by making use of embryoid body cultures from mouse embryonic stem cells (ESCs). In this model, stable FHL2 gain-of-function promoted mesodermal cell formation and cell proliferation while arresting cardiac differentiation in an early cardiogenic mesodermal progenitor state. Mechanistically, FHL2 overexpression enhanced nuclear accumulation of β-catenin and activated Wnt/β-catenin-dependent transcription leading to sustained upregulation of the early cardiogenic gene Igfbp5. In an alternative P19 cell model, transient FHL2 overexpression led to early activation of Wnt/β-catenin-dependent transcription, but not sustained high-level of Igfbp5 expression. This resulted in enhanced cardiogenesis. We propose that early Wnt/β-catenin-dependent transcriptional activation mediated by FHL2 is important for the transition to and expansion of early cardiogenic mesodermal cells. Collectively, our findings offer mechanistic insight into the early cardiogenic code and may be further exploited to enhance cardiac progenitor cell activity in vitro and in vivo. Stem Cells *2013;31:928–940*

## INTRODUCTION

The extensively described canonical Wnt signaling pathway has as a key mediator, the ubiquitously expressed protein β-catenin, which plays a dual role in cell adhesion and signal transduction [1]. In absence of Wnt, cytosolic β-catenin is recruited to the destruction complex composed of Axin, adenomatous polyposis coli 2, and glycogen synthase kinase 3 (GSK-3), which leads to GSK-3-mediated phosphorylation and subsequently proteasomal degradation of β-catenin [2]. In contrast, Wnt binding to its receptor complex, composed by members of the Frizzled receptors and low density lipoprotein receptor-related protein, results in the dissociation of the β-catenin destruction complex, which prevents GSK-3-mediated phosphorylation. This leads to cytoplasmic accumulation of β-catenin and subsequently translocation into the nucleus, where it binds to the lymphoid enhancer-binding factor (LEF)/T-cell specific, HMG-box (TCF) cofactors and activates target gene transcription [3]. Temporal control of canonical Wnt signaling is essential for normal gastrulation and subsequently germ layer segregation in the early epiblast [4, 5]. Later during development, the canonical Wnts promote cardiogenesis during mesoderm induction, but act as inhibitors of committed cardiac progenitor cells in vivo and in vitro [6, 7]. Tight control of Wnt-mediated gene expression is critical for the regulation of cell-specific downstream effectors and essential for cardiac cell development. Although the role of the canonical Wnt pathway in cardiogenesis is now explained in a highly complex multiphasic model, the precise regulation of this pathway remains to be investigated.

Several studies suggest that members of the LIM-only protein family may act as coregulators of tissue-specific gene expression by interacting with different transcription factors. Specifically, the four-and-a-half-LIM-domain (FHL) 2 was shown to interact with β-catenin in myoblast and tumor cell lines and to regulate its transcriptional activity [8, 9]. FHL2 was initially identified as downregulated in rhabdomyosarcomas LIM domain protein and characterized for its abundant expression in the human heart [10]. FHL2 contains a cysteine-rich consensus sequence, which is known as a potent protein-protein interaction motif regulating a variety of cell processes [11]. Through different proteins-interactions, FHL2 functions as a signal transducer/transmitter in different subcellular compartments and potentially also as a transcription regulator [11, 12]. Despite the strong expression of FHL2 in the heart, the role of the protein in cardiac cells remains unclear. Notably, FHL2-deficient mice do not show a spontaneous cardiac phenotype [13]. This may at least in part be due to compensatory mechanisms via structurally similar FHL-protein family members, such as FHL1 and FHL3 [13–15]. However, β-adrenergic stimulation resulted in exacerbated dilated cardiomyopathy in FHL2-deficient mice [13]. This may be explained by the role of FHL2 in coupling titin and calcineurin activity to metabolic enzymes [16, 17]. Currently, no data are available concerning the transcriptional role of FHL2 in cardiomyocyte development.

Given the role of β-catenin in the context of the Wnt canonical signaling in early and adult cardiogenesis, we were interested in identifying novel cardiac-specific Wnt/β-catenin regulators. Using a yeast-two-hybrid (Y2H) screen, we found in this study an interaction between FHL2 and β-catenin, resulting in an activation of the β-catenin-dependent transcription in cardiac cells. To explore the role of this interaction in cardiogenesis, we used embryonic stem cell (ESCs) and embryonic carcinoma cell (P19) embryoid body (EB) cultures, which can consistently recapitulate the sequences of cardiac genes expression observed in early cardiac development in the mouse embryo [18]. We observed that stable FHL2 gain-of-function in ESC-EBs promoted mesodermal cell formation and abrogated cardiac differentiation by locking cardiac mesodermal cells in a progenitor state. Moreover, transient overexpression of FHL2 in P19-EBs led to enhanced cardiac progenitor cell activity with subsequent augmentation of cardiomyocyte differentiation. Collectively, our study provides evidences for a novel role for FHL2-mediated β-catenin regulation in early cardiogenesis.

## MATERIALS AND METHODS

### Yeast-Two-Hybrid

The ProQuest Two-Hybrid System (Invitrogen Germany, Darmstadt, Germany, http://www.invitrogen.com) was used as described previously [19]. The pDBLeu-β-catenin GAL4-DNA-binding domain containing plasmid was used as bait to screen against a human cardiac cDNA library (kindly provided by Prof. N. Frey, Kiel, Germany).

### Southern Blot

Genomic DNA was purified using the DNeasy Tissue Kit (Qiagen Germany, Hilden, Germany, http://www.qiagen.com); digested with EcoRI, BamHI, and XhoI restriction enzymes, electrophoresed, and transferred onto a nitrocellular membrane (Stratagene Germany, Waldbronn, Germany, http://www.genomics.agilent.com). The riboprobes were generated by ClaI digestion of the cmyc-*Fhl2* plasmid (kindly provided by Dr. V. Wixler, Münster, Germany) and Digoxigenin (DIG)-labeling (Roche Germany, Mannheim, Germany, http://www.roche-applied-science.com). Probes were hybridized overnight at 68°C; membranes were washed at 68°C in low- and high-stringency buffer (0.5× Saline-sodium citrate (SSC)/0.1% SDS; 2× SSC/0.1% SDS). Detection was performed with an anti-DIG antibody and CPC-star (Roche) [20].

### Cell Culture

Double transgenic αMHC-GFP/αMHC-Neomycin resistance cassette sequence (NeoR) murine ESCs were transfected on Matrigel (BD Bioscience Germany, Heidelberg, Germany, http://www.bdbiosciences.com) with the cmyc-*Fhl2* expression plasmid and a puromycin-expressing vector using the Xfect-stem reagent (Clontech Takara Bio Europe, Saint-Germain-en-Laye, France, http://www.clontech.com). Transfected cells were selected under puromycin (1 μg/ml). For differentiation, cells were aggregated in hanging drops containing 500 cells each to form EBs and cultured for 5 days in Iscove medium supplemented with 20% fetal calf serum (FCS) and 0.1 mM ascorbic acid as previously described [21]. After 5 days, cells were plated on 0.1% gelatin-coated dishes and cultured. At day 11 of differentiation, cardiomyocytes cells were selected with the neomycin derivate G418 Invitrogen (200 μg/ml). αMHC-GFP-expression was documented using an IX70 Olympus microscope. EBs containing beating areas were counted and presented in percent of total EBs. For rescue experiments, differentiating ESCs were treated with 5 μmol/l quercetin (Acros Organics Belgium, Geel, Belgium, http://www.acros.be) or dimethylsulfoxid (DMSO) at the indicated time points.

P19 were transfected with a cmyc-*Fhl2*-expressing as well as an empty vector and cultured as previously described [22]. For differentiation, cells were aggregated 48 hours post-transfection in hanging drops containing 400 cells for 2 days and cultured in suspension for additional 3 days in Dulbecco's modified Eagle's medium-F-12 medium containing 20% FCS and 1% DMSO. Aggregates were plated onto 0.1%-gelatin-coated 12-well-plates and coverslips for analysis.

Neonatal rat cardiomyocytes (NRCMs) were prepared as described previously [23]. Briefly, NRCMs were isolated from 2 to 3-day-old Wistar rats (Charles River Germany, Sulzfeld, Germany, http://www.criver.com), digested with collagenase type II (Worthington Biochemicals U.S., Lakewood, U.S., http://www.worthington-biochem.com), purified by discontinuous Percoll gradient centrifugation, plated on gelatin, and cultured. Transfection was performed using FuGene (Roche) according to manufacturer's instruction.

### Immunofluorescence

ESCs were differentiated on 0.1% gelatin-coated coverslips. Cells were fixed in 4% paraformaldehyd (PFA), permeabilized in 0.1% Triton X-100, and blocked in 1% bovine serum albumin (BSA). Cells were incubated with respective antibodies against β-catenin (BD Bioscience); α-sarcomeric actinin (Sigma-Aldrich Germany, Hamburg, Germany, http://www.sigmaaldrich.com); FHL2, cardiac troponin T (cTNT), Igfbp5, and Ki67 (Abcam U.K., Cambridge, U.K., http://www.abcam.com); 1:200 dilutions. AlexaFluor488- and AlexaFluor594-conjugated secondary antibodies (1:200; Invitrogen) were used for labeling. For nuclei visualization, cells were stained with 4′,6-diamidino-2-phenylindole and mounted with ProlongGold (Invitrogen). Microscopic images were captured with a confocal microscope (Zeiss LSM710/NLO). Semiquantitative analysis was performed using the Axiovision software (Zeiss Germany, Jena, Germany, http://www.zeiss.de).

### Luciferase Reporter Assay

NRCMs were cotransfected with FHL2- and/or nondegradable β-catenin-expressing plasmids along with the pTOPflash luciferase reporter and *Renilla* luciferase-expressing plasmid for normalization. pFOPflash containing mutated TCF binding sites was used as negative control. Luciferase activity was determined using dual-luciferase reporter assay (Promega U.S., Madison, U.S., http://www.promega.com) 48 hours after transfection, according to manufacturer's instructions.

### Flow Cytometry Analysis

Cells were fixed in 1% formaldehyde/phosphate buffered saline (PBS), permeabilized in flow cytometry buffer containing 0.5% Saponin (Sigma-Aldrich), and stained with antibodies directed against α-sarcomeric actinin (1:200; Sigma-Aldrich) and Nkx2.5 (1:200; Santa Cruz Biotechnology U.S., Dallas, U.S., http://www.scbt.com). Cells were stained with anti-rabbit IgG-APC or anti-mouse F(ab)_2_-FITC (1:500; Jackson Immuno Research U.K., Newmarket, U.K., http://www.jacksonimmuno. com). Respective isotype controls were used. Fluorescence signals were detected with a Calibur flow cytometer (BD).

### RNA Isolation, Reverse Transcription, and Quantitative Real-Time PCR Analysis

Total RNA was isolated from cells, embryonic, and postnatal tissue using the RNA II kit (Macherey-Nagel Germany, Düren, Germany, http://www.mn-net.com). cDNA was synthesized and quantitative real-time PCR analyses were performed with SYBR Green (Qiagen) on an iCycler instrument (BioRad Germany, Munich, Germany, http://www.biorad.com). Copy numbers were calculated using the iCycler software with a relative standard curve obtained using the log dilutions of gene of interest cDNA. All reactions were run in triplicates and normalized to gapdh. Primers are listed in supporting information Table S1.

### Coimmunoprecipitation and Immunoblotting

Forty-eight hours after transfection, cells were harvested, lysed with Baeuerle buffer with protease inhibitors, and immunoprecipitated with an anti-β-catenin antibody (Santa Cruz). Detection was done by immunoblotting using a c-myc (Santa Cruz) antibody. Protein lysate transfected with empty vector served as control. Whole cell lysates were immunoblotted with respective antibodies to detected protein expression. For Western blot anti-FHL2 (Abcam), anti-β-catenin (Santa Cruz) and anti-GAPDH (Zytomed System Germany, Berlin, Germany, http://www.zytomed-systems.de) antibodies were used. Densitometric analysis was performed using Adobe Photoshop software.

### Statistical Analysis

Differences between experimental groups were analyzed using two-tailed Student's *t* test or ANOVA test followed by Bonferroni's multiple comparison test. Data are presented as mean ± SEM. *p* < .05 values were considered significant.

## RESULTS

### Identification of FHL2 as a Novel Regulator of β-Catenin-Dependent Transcription in Cardiac Cells

**Figure 1:**
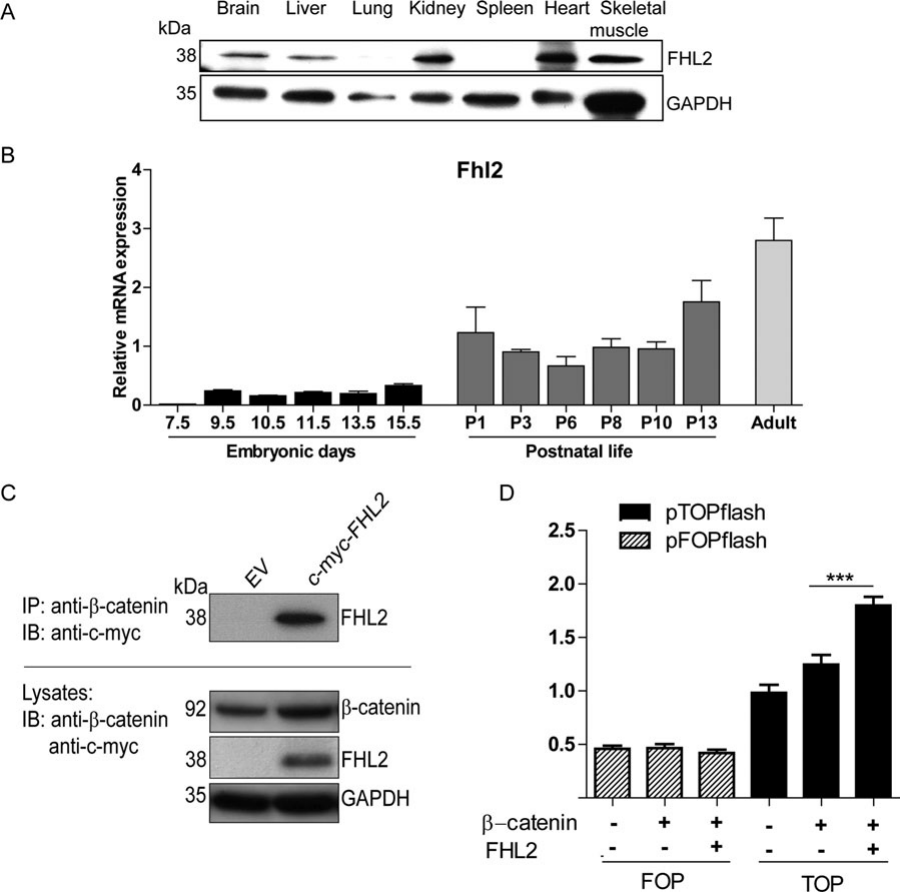
FHL2 is expressed in cardiac tissue, interacts with β-catenin, and enhances its transcriptional activity in heart cells. **(A):** Protein expression analysis of FHL2 via Western blot in different adult organs in mice normalized to GAPDH. **(B):** qPCR analysis of Fhl2 in embryonic cardiac developing tissue, early postnatal, and adult mouse heart. Relative mRNA levels were normalized to tumor protein, translationally controlled 1 (Tpt1), a gene expressed with insignificant variation along development and adulthood. **(C):** FHL2 and β-catenin interact in neonatal rat cardiomyocyte (NRCM) cultures. NRCM cultures were transfected with a c-myc-FHL2-expressing plasmid and an EV (as control), subsequently endogenous β-catenin was co-IP and FHL2 was IB with a c-myc-antibody. Both proteins were also detected in protein lysates from NRCM cultures. **(D):** FHL2 activates β-catenin-dependent transcription in NRCM cultures. Luciferase activity measurement demonstrated significant activation of the pTOPflash luciferase LEF/TCF reporter activity upon FHL2 and β-catenin coexpression in NRCM. The FOPflash-plasmid containing mutated TCF binding sites served as control and Renilla luciferase was used for normalization. Data represent mean ± SEM, *n* = 3, ***, *p* < .001 (ANOVA; Bonferroni's multiple comparison test). Abbreviations: EV, empty vector; FHL2, four-and-a-half-LIM-domain 2; IB, immunodetected; IP, immunoprecipitated.

A Y2H system using a human cardiac expression library was used to screen cardiac β-catenin binding proteins [24]. From 315 isolated clones, 26 clones containing inserts corresponding to the coding sequence for FHL2 were identified as specific β-catenin interaction partners. None of these 26 clones showed an interaction with the GAL4-DNA-binding domain alone indicating a specific binding to β-catenin in yeast (data not shown). To corroborate the relevance of FHL2 in the cardiovascular system, expression analysis of FHL2 was performed in different adult organs. In agreement with previous reports, we observed prominent expression of FHL2 protein in adult heart, skeletal muscle, and kidney (Fig. 1A); lower protein abundance was found in brain and liver. Comparative analysis in embryonic, fetal, and postnatal cardiac tissue showed Fhl2 transcript expression as early as embryonic day (ED) 7.5 with continuously increasing transcript abundance until adulthood (Fig. 1B). The interaction between β-catenin and FHL2 was validated by coimmunoprecipitation in NRCM cultures transfected with a cmyc-*Fhl2* plasmid (Fig. 1C). Endogenous β-catenin was immunoprecipitated with a β-catenin antibody and FHL2 were detected with a specific c-myc antibody. This confirmed the interaction between β-catenin and FHL2 in cardiac mammalian cells. To further examine the functional role of this interaction, NRCM cultures were transfected with the pTOPflash reporter plasmid, which is activated when β-catenin binds to LEF/TCF elements driving firefly luciferase expression. As expected, the TOPflash reporter gene activity was enhanced by endogenous and cotransfected β-catenin. When FHL2 was coexpressed with β-catenin, the luciferase activity was further enhanced (Fig. 1D, *p* < .001, *n* = 3/group). Altogether these data indicate that FHL2 binds β-catenin in cardiac cells to exert a synergistic activity on β-catenin transcriptional activity.

### FHL2 Stimulates β-Catenin-Dependent Transcriptional Activation in ESC-EB Culture

**Figure 2:**
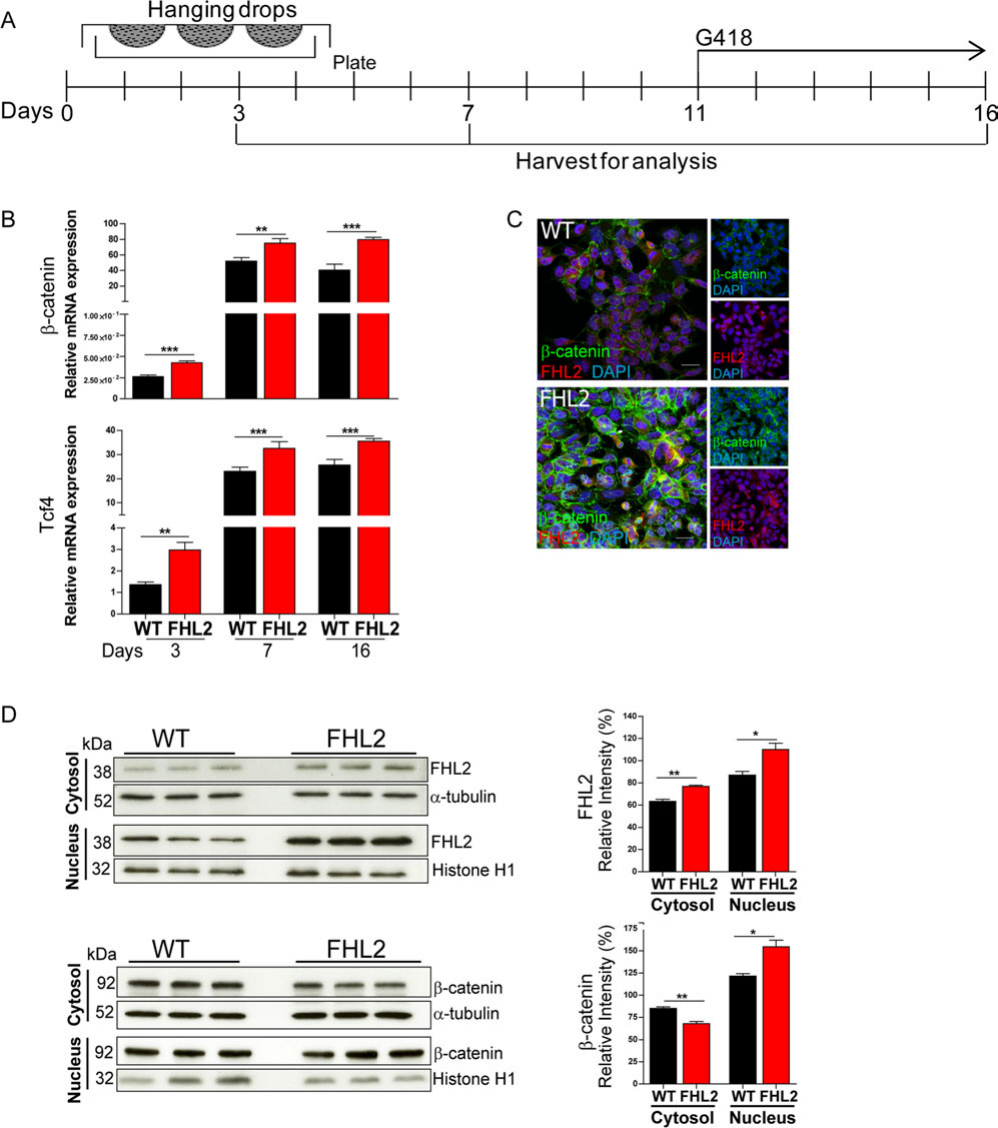
Nuclear accumulation of β-catenin and enhanced transcriptional activity mediated by FHL2 in mouse embryonic stem cell-embryoid body cultures (ESC-EBs). **(A):** Schematic representation of the 16 days differentiation protocol for in vitro cardiogenesis using ESCs. Differentiating ESCs were harvested for analysis following 3, 7, and 16 days of differentiation. At day 11 of differentiation, αMHC cells were selected with the neomycin derivate G418. **(B):** β-Catenin/TCF transcriptional activation under FHL2 gain-of-function. Significantly increased expression of β-catenin and Tcf4 following 3, 7, and 16 days as revealed by qPCR in ESC-EBs. mRNA levels are displayed as relative expression to Gapdh. **(C):** Confocal immunofluorescence analysis showing upregulated β-catenin signal (green) and FHL2 (red) in undifferentiated ESC-overexpressing FHL2. DAPI (blue) was used for nuclear staining. **(D):** Nuclear β-catenin accumulation upon FHL2 gain-of-function. Immunoblot showing increased FHL2 expression as well as β-catenin nuclear accumulation along with cytosolic depletion in FHL2-ESCs. Semiquantitative assessment via densitometry in three independent experiments. Normalization was performed with α-tubulin for the cytosolic and Histone H1 for the nuclear fraction. Data represent mean ± SEM; (B): *n* = 6; (D): *n* = 3, *, *p* < .05; **, *p* < .01; ***, *p* < .001 (two-tailed Student's *t* test). Scale bar = 20 μm. Abbreviations: DAPI, 4′,6-diamidino-2-phenylindole; FHL2, four-and-a-half-LIM-domain 2.

Next, we tested the hypothesis that FHL2 and β-catenin interaction and regulation play a role during cardiogenesis in ESC-EB cultures (Fig. 2A). Double transgenic ESCs harboring a green fluorescent protein (GFP) reporter as well as a neomycin resistance driven by the cardiomyocyte-restricted αMHC promoter (αMHC-GFP/αMHC-Neo-ESCs) were used in this study. First, αMHC-GFP/αMHC-Neo-ESCs stably expressing c-myc-tagged recombinant FHL2 (FHL2-ESCs) were generated (supporting information Fig. S1). Three positive clones were selected for verification of transgene integration by Southern blotting (supporting information Fig. S1A, S1B); clone 8 was selected for further analysis. Overexpression of FHL2 was confirmed by quantitative PCR (supporting information Fig. S1C) and Western blot analyses (supporting information Fig. S1D). Gene expression analysis revealed an upregulation of β-catenin as well as its transcriptional cofactor Tcf4 in FHL2-ESC-line #8-EB cultures compared to wild-type ESC-EBs at 3, 7, and 16 days of differentiation (*n* = 6/group, Fig. 2B). Confocal immunofluorescence analysis confirmed increased β-catenin expression in FHL2-ESC (Fig. 2C). Immunoblots of subcellular fractions documented enhanced abundance of FHL2 in the cytosolic and nuclear compartments of the transgenic FHL2-ESC cells, while β-catenin was elevated in the nuclear lysate, but concomitantly reduced in the cytosolic fraction (*n* = 3/group, Fig. 2D). These data collectively confirm that nuclear accumulation of β-catenin in FHL2-overexpressing cells enhances transcriptional activity of the Wnt/β-catenin/TCF4 cascade in ESCs.

### Overexpression of FHL2 Blocks Cardiac Differentiation at the Cardiac Progenitor Stage

**Figure 3:**
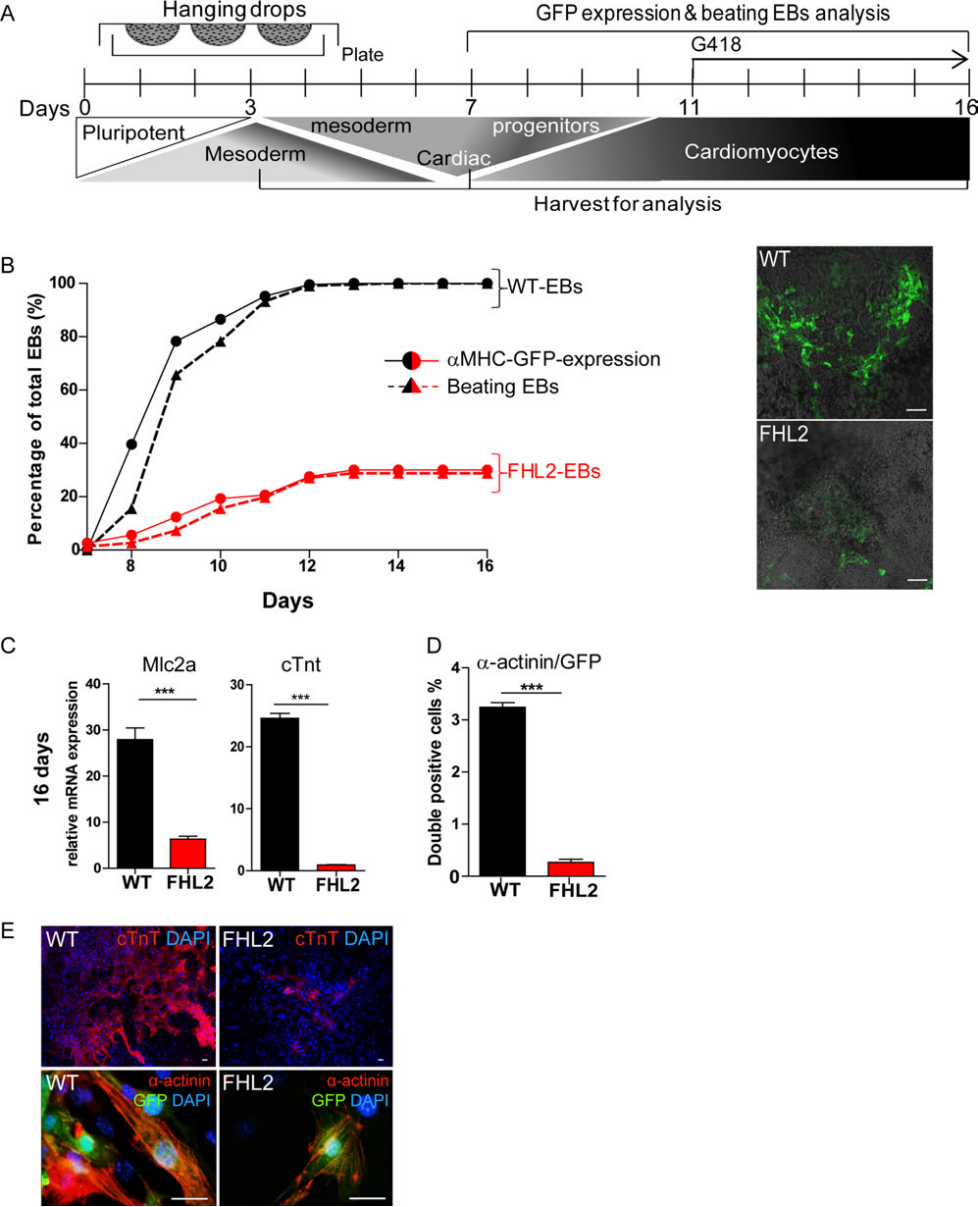
FHL2 gain-of-function blocks cardiac differentiation in embryonic stem cell (ESC)-EBs. **(A):** Schematic representation of the 16-day differentiation protocol for in vitro cardiogenesis. Differentiating ESC-EBs were harvested for analysis on culture days 3, 7, and 16. **(B):** ESC-EBs overexpressing FHL2 showed decreased cardiac differentiation. Beating EBs (triangles and discontinues lines) and αMHC-GFP expression (dots and continuous lines) were measured as percentage of total EBs. Both parameters were significantly reduced in FHL2- (red lines) in comparison to wild-type ESC-EBs (black lines) during cardiac differentiation in vitro. Representative immunofluorescence pictures showing αMHC-GFP expression in both cell lines are shown (right). **(C):** Reduced gene expression of cardiomyocytes genes in ESC-EBs overexpressing FHL2. FHL2-ESC-EBs showed significant reduction in myosin light chain 2a and cardiac Troponin T expression as measured by qPCR; mRNA levels are normalized to Gapdh. **(D):** Reduction in α-sarcomeric actinin (α-actinin)/GFP cell number as measured by flow cytometry following 16 days of differentiation. **(E):** Representative immunofluorescence pictures with lower cTNT and α-actinin/αMHC-GFP expression in FHL2- in comparison to wild-type-ESC-EBs. DAPI (blue) was used for nuclear staining. Data represent mean ± SEM; (B): *n* = 12; (C): *n* = 6, (D): *n* = 3, ***, *p* < .001 (two-tailed Student's *t* test). Scale bar = (B): 100 μm and (E): 20 μm. Abbreviations: DAPI, 4′,6-diamidino-2-phenylindole; EB, embryoid body; FHL2, four-and-a-half-LIM-domain 2; GFP, green fluorescent protein.

We further tested the functional relevance of FHL2-dependent regulation of β-catenin transcriptional activity during cardiogenesis in ESC-EB culture (Fig. 3A). Formation of cardiomyocytes can be readily identified and approximated by enumeration of spontaneous contracting EBs. In addition, cardiomyocyte-restricted GFP-expression (αMHC-GFP) was used to quantify cardiomyocyte formation. Direct comparison of transgenic versus wild-type ESC-EBs demonstrated a markedly reduced cardiogenicity in FHL2-overexpressing ESC-EBs (*n* = 12/group, Fig. 3B). This was further substantiated by the observation of reduced expression of cardiomyocyte-specific genes (myosin light chain [Mlc] 2a and cTnt) at day 16 of differentiation (Fig. 3C; *n* = 6/group). Similarly, α-sarcomeric actinin/GFP-positive cells as detected by flow cytometry (*n* = 3/group, Fig. 3D) and cTNT expression as detected by immunofluorescence (Fig. 3E) were markedly decreased in FHL2-overexpressing versus wild-type ESC-EBs on culture day 16. These data suggest an abrogation of the cardiac differentiation program upon stable FHL2 overexpression.

**Figure 4:**
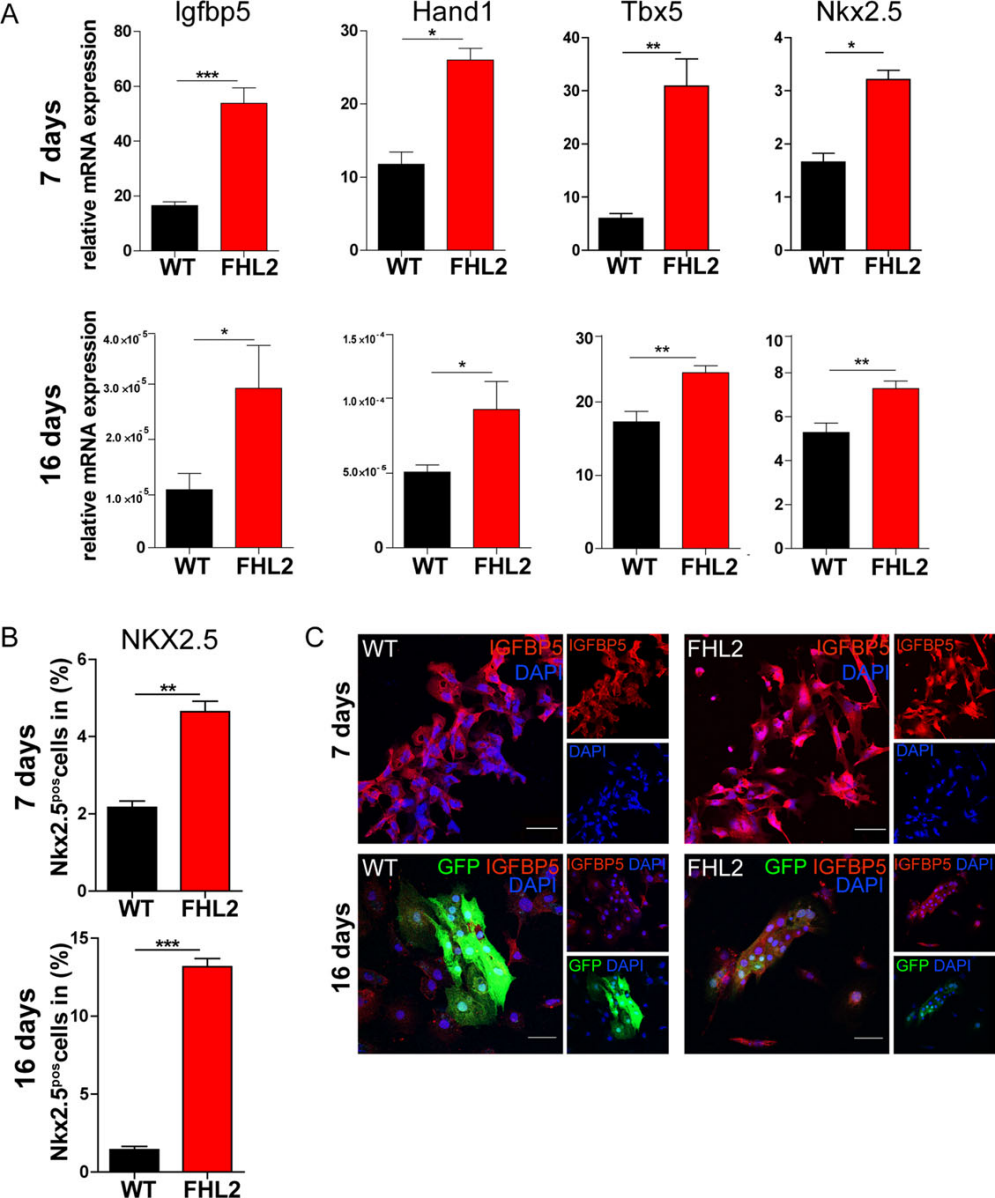
FHL2 mediates enhanced activation of early cardiogenic gene expression in embryonic stem cell-embryoid bodies (ESC-EBs). **(A):** Expression of the early cardiogenic lineage genes Igfbp5, Hand1, Nkx2.5, and Tbx5 following 7 and 16 days of differentiation upon FHL2 gain-of-function in ESC-EBs as measured by qPCR; mRNA levels are normalized to Gapdh. **(B):** FHL2 gain-of-function in ESC-EBs resulted in an augmented early cardiogenic cell population as revealed by increased NKX2.5 cell number analyzed by flow cytometry on culture days 7 and 16. **(C):** Sustained upregulated IGFBP5 expression in FHL2-ESC-EBs accompanied by decreased αMHC-GFP-expressing cells. Increased nuclear localized IGFBP5 expression at 7 days of differentiation in FHL2-ESC-EBs is observed via confocal immunofluorescence analysis. Increased IGFBP5 along with decreased αMHC-GFP expression detected at 16 days of differentiation in FHL2-ESC-EBs. DAPI (blue) was used for nuclear staining. Data represent mean ± SEM; (A): *n* = 12, (B): *n* = 3, *, *p* < .05; **, *p* < .01; ***, *p* < .001 (two-tailed Student's *t* test). Scale bar = 50 μm. Abbreviations: DAPI, 4′,6-diamidino-2-phenylindole; FHL2, four-and-a-half-LIM-domain 2; GFP, green fluorescent protein.

Since FHL2 was reported to be necessary for regulating skeletal myoblast cell populations [8, 25], we next analyzed whether FHL2 would also regulate early cardiac gene activation during ESCs differentiation. At day 7 of differentiation, higher gene expression of early cardiogenic progenitor markers such as insulin growth factor binding protein 5 (Igfbp5), Hand1, Tbx5, and Nkx2.5 was observed in FHL2-overexpressing versus wild-type ESC-EBs (*n* = 12/group; Fig. 4A). Moreover, a continuously elevated transcript abundance of these markers was detected also at day 16 of ESC-EB culture, despite the anticipated downregulation of Igfbp5 and hand1in differentiating ESC-EBs. Higher NXK2.5 cell number in FHL2 versus wild-type ESC-EBs was also confirmed by flow cytometry at day 7 and 16 of differentiation (Fig. 4B). Interestingly, IGFBP5 protein expression in the FHL2-overexpressing ESC-EBs appeared to be predominantly localized in the nuclear compartment as shown by confocal immunofluorescence analysis at 7 days of differentiation (Fig. 4C, upper panels). On day 16, FHL2-ESC-EBs showed pronounced IGFBP5 and less GFP expression, in contrast to wild-type-ESC-EBs showing prominent GFP and lower IGFBP5 expression (4C, lower panels). Collectively, these results indicate that expression of FHL2 blocks cardiac differentiation by arresting the cells in an early progenitor stage.

### FHL2 Regulates Stem Cell Lineage Commitment and Cell Proliferation in ESC-EB Cultures

**Figure 5:**
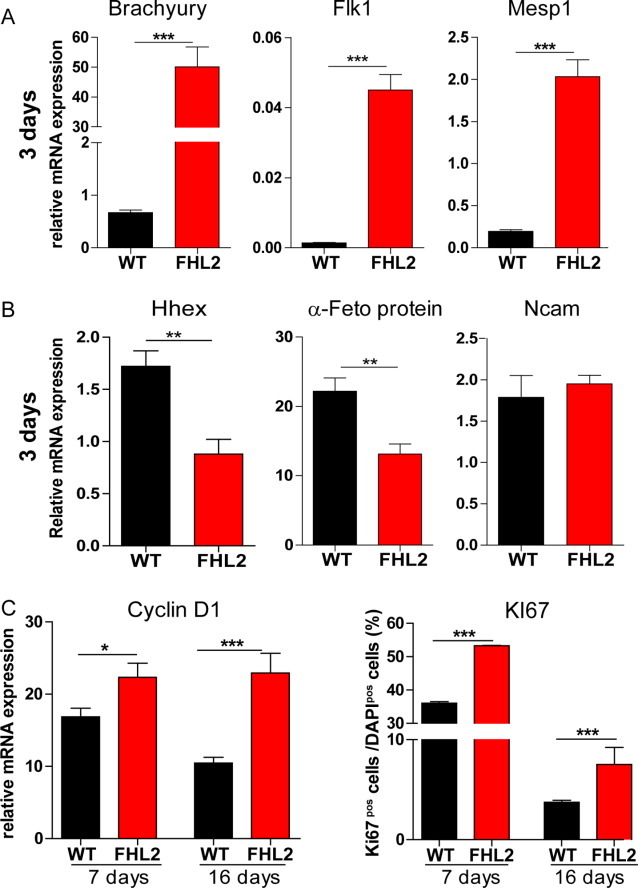
FHL2 gain-of-function promotes mesodermal cell specification and proliferation in embryonic stem cell-embryoid bodies (ESC-EBs). **(A):** At day 3 of differentiation, FHL2 gain-of-function in ESC-EBs increases expression of the early panmesodermal, mesodermal, and cardiac mesodermal markers Brachyury, Flk1, and Mesp1, respectively, as shown by qPCR analysis. **(B):** FHL2-ESC-EBs showed decreased expression of the endodermal markers Hhex and α-Feto protein; no change in expression of the neuroectodermal marker Ncam was observed in comparison to wild-type-ESC-EBs at day 3 of differentiation. **(C):** FHL2 overexpression promoted significantly augmented expression of the cell cycling marker Cyclin D1 as shown by qPCR as well as increased Ki67 expression as demonstrated by immunofluorescence microscopy (percentage of total DAPI-positive cells) following 7 and 16 days of differentiation. mRNA level was normalized to Gapdh. DAPI (blue) was used for nuclear staining. Data represent mean ± SEM; *n* = 6, *, *p* < .05; **, *p* < .01; ***, *p* < .001 (two-tailed Student's *t* test). Abbreviation: FHL2, four-and-a-half-LIM-domain 2.

Since activation of the Wnt signaling pathway is important for establishment of early mesodermal cells, which give rise to cardiac cells, we hypothesized that FHL2-dependent β-catenin transcriptional activation would stimulate mesodermal cell lineages commitment. Therefore, we first examined changes in gene expression of differentiation markers at day 3 of ESCs-differentiation. This analysis revealed significant activation of the panmesodermal marker T-box transcription factor brachyury along with the early more restricted mesodermal receptor tyrosine kinase Flk1 and the earliest cardiac marker basic helix-loop-helix transcription factor mesoderm posterior 1 (Mesp1) in FHL2- versus wild-type-ESC-EBs (*n* = 6/group, Fig. 5A). In contrast, the expression of the endodermal markers Hhex and α-Feto protein was significantly reduced, whereas the neuroectodermal marker neural cell adhesion molecule (Ncam) was unchanged in FHL2-overexpressing ESCs in comparison to WT (*n* = 6/group, Fig. 5B). Gene expression analysis at 7 days of differentiation showed significant lower expression of the pluripotent marker Oct4 as well as Brachyury and Mesp1 upon FHL2 gain-of-function (supporting information Fig. S2).

Since FHL2 was reported to have a role in cell cycle regulation in noncardiac cell types [26], we compared surrogate markers for cell proliferation in FHL2-overexpressing and wild-type ESC-EB cultures. Interestingly, expression of the G1/S-specific gene Cyclin D1, detected by qPCR, as well as cells expressing the proliferation marker KI67, detected by immunofluorescence, was significantly increased at days 7 and 16 of cardiac differentiation in FHL2-ESC-EB cultures (*n* = 6/group, Fig. 5C). Collectively, these data indicate that FHL2 stimulates formation and expansion of early mesodermal cells at the expense of endodermal cells.

### Blockage of β-Catenin-Dependent Transcriptional Activation After Mesoderm Formation Rescues the FHL2-Dependent Inhibition of Cardiac Differentiation in ESCs

**Figure 6:**
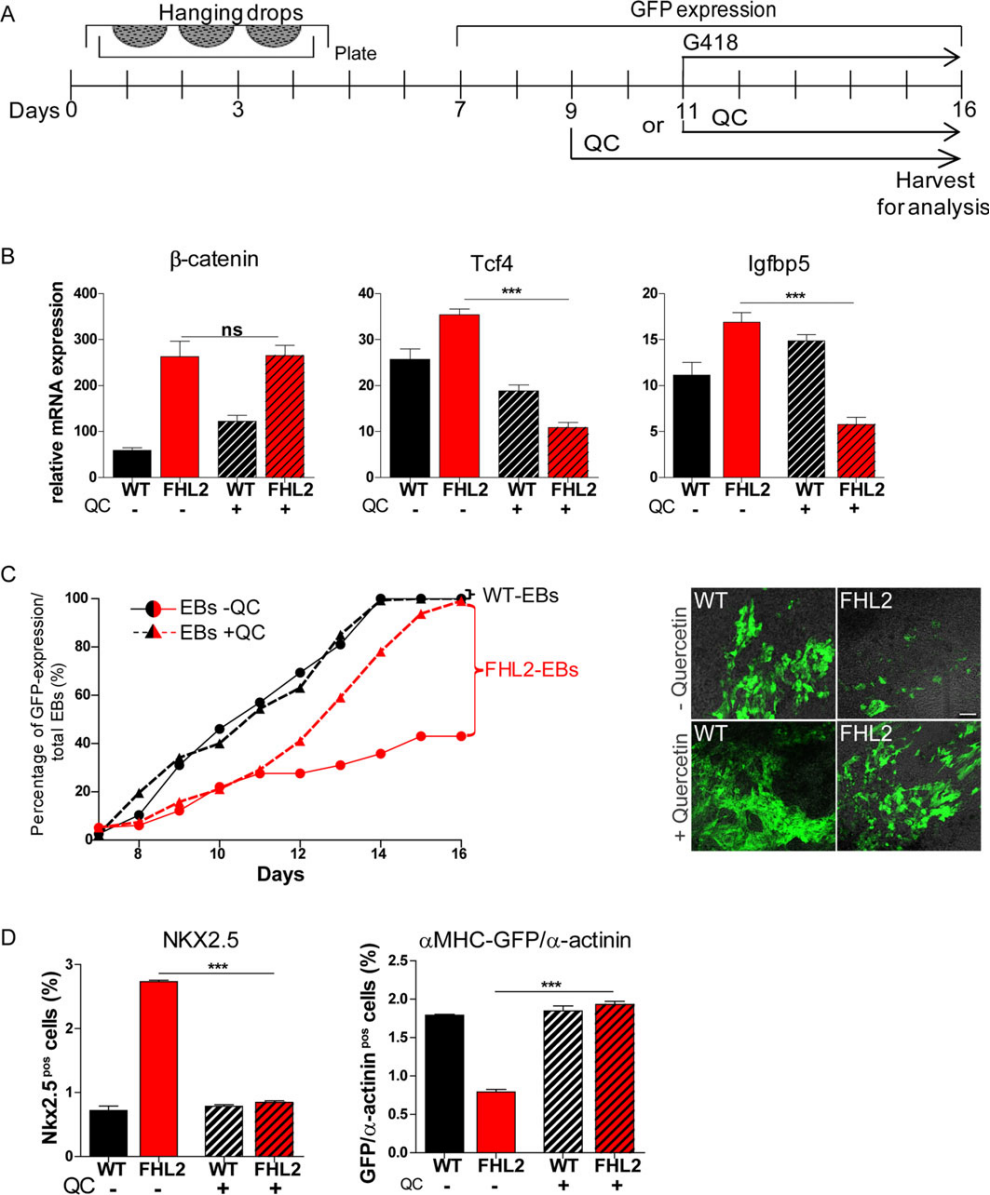
Inhibition of β-catenin-dependent transcription rescues cardiac differentiation in FHL2-ESC-EBs. **(A):** Schematic representation of in vitro cardiogenesis in embryonic stem cell (ESC)-EBs upon 5 μmol/l QC application from day 9 or 11 of differentiation. Cells were harvested at day 16 of differentiation for analysis. **(B):** Transcriptional effect of quercetin treatment on differentiating FHL2-ESCs. qPCR analysis showed unchanged β-catenin expression but reduced levels of upregulated Tcf4 and Igfbp5 expression in FHL2-ESC-EBs upon quercetin treatment in comparison to untreated FHL2-ESC-EBs following 16 days of differentiation. mRNA levels were normalized to Gapdh. **(C):** Blocking β-catenin transcriptional upregulation via quercetin increased the expression of cardiomyocyte genes in FHL2-ESC-EBs. Quercetin treatment at day 11 reversed the decreased αMHC-GFP expression during cardiac differentiation in FHL2-ESC-EBs (triangles and discontinues red lines) in comparison to untreated FHL2-ESC-EBs (dots and continues red lines) expressed as percentages of total EBs. Quercetin did not affect expression of αMHC-GFP in wild-type-ESC-EBs (black lines). Representative confocal immunofluorescence pictures of the αMHC-GFP expression are shown (right). **(D):** Flow cytometry shows a rescue in upregulation of NKX2.5 as well as in downregulation of α-MHC-GFP/α-sarcomeric-actinin-expressing cells in FHL2-ESC-EBs compared to untreated FHL2-ESC-EBs at 16 days of differentiation. Data represent mean ± SEM; (B): *n* = 6, (C): *n* = 10, (D): *n* = 3, ***, *p* < .001 (ANOVA; Bonferroni's multiple comparison test). Scale bar = 100 μm. Abbreviations: EB, embryoid body; FHL2, four-and-a-half-LIM-domain 2; GFP, green fluorescent protein; QC, quercetin.

We found activation of the β-catenin-dependent transcription upon FHL2 overexpression, which is known to block differentiation of cardiomyogenic cells [7]. Therefore, we asked whether the prevention of this transcriptional activation after mesodermal specification would result in terminal cardiac differentiation of ESCs overexpressing FHL2. To answer this question, we inhibited β-catenin-dependent transcription with quercetin, a known pharmacological inhibitor of β-catenin/Transcription factor 7-like 2 (TCF7L2 or TCF4) signaling [27]. Quercetin (5 μM) was applied to FHL2- and wild-type-ESC-EB cultures from differentiation days 9 and 11; this was followed by an analysis of cardiac differentiation on culture day 16 (Fig. 6A). qPCR showed no change in the FHL2-dependent β-catenin transcript upregulation while Tcf4 was reduced under quercetin, as expected (*n* = 6/group, Fig. 6B). Moreover, Igfbp5 gene expression was reduced under quercetin on FHL2-ESC-EB culture day 16 (Fig. 6B). Accordingly, cardiomyocyte differentiation was rescued under inhibition of β-catenin-dependent transcription by quercetin in FHL2-ESC-EB cultures (*n* = 12/group, Fig. 6C; supporting information Fig. S3). Furthermore, the observed increase in NKX2.5-expressing cells as well as the reduced cardiomyocyte number, based on GFP and α-actinin expression measured by flow cytometry, was normalized upon quercetin treatment (*n* = 3/group, Fig. 6D). These findings confirm that high expression of β-catenin induced by FHL2 mediates the final cardiac differentiation arrest in ESCs overexpressing FHL2.

### Transient FHL2 Expression Promotes Cardiac Cell Formation in P19 Cells In Vitro

**Figure 7:**
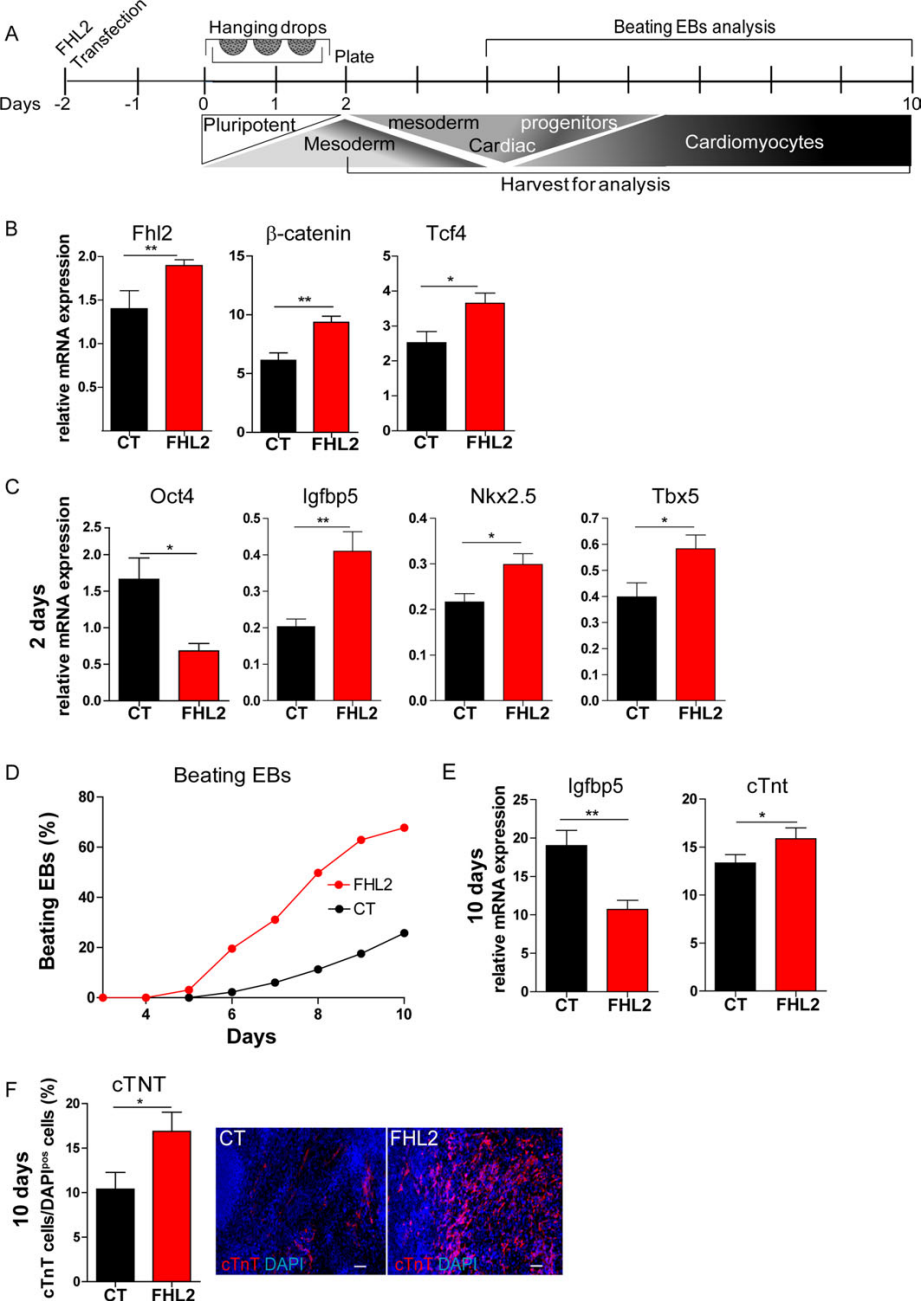
Transient overexpression of FHL2 enhances cardiogenesis in P19 cells in vitro. **(A):** Schematic representation of differentiation protocol for in vitro cardiogenesis. P19 cells were differentiated in the presence of Dimethyl sulfoxide (DMSO) and harvested for analysis following 1, 2, and 10 days of differentiation. **(B):** Transient upregulation of FHL2, β-catenin, and its target gene Tcf4 in P19 cells transfected with a FHL2-expressing plasmid (FHL2) as demonstrated by qPCR analysis 24 hours post-transfection. **(C):** Increased early cardiogenic commitment in FHL2-P19-EBs transiently expressing cells. Significant loss of the pluripotent marker Oct4 and increase of the early cardiogenic markers Igfbp5, Nkx2.5, and Tbx5 following 2 days of differentiation in FHL2-EBs compared to control-EBs (CT) as shown by qPCR analysis. mRNA levels were normalized to Gapdh. **(D):** Increased cardiogenesis in P19-EBs transiently expressing FHL2. Augmentation of beating EB numbers was observed along cardiac differentiation of FHL2-P19 cells in comparison to CT. **(E):** Significant downregulation of Igfbp5 expression and augmentation of cTnt expression normalized to Gapdh was observed in FHL2-P19-EBs following 10 days of differentiation. **(F):** cTNT (red) expression analyzed by immunofluorescence microscopy following 10 days of differentiation showing increased cTNT cell number (as percentage of total DAPI-positive cells) in FHL2-P19 cells. Representative pictures are depicted. DAPI (blue) was used for nuclear staining. Data represent mean ± SEM; B, C, E, and F: *n* = 6, D: *n* = 9, *, *p* < .05; **, *p* < .01 (two-tailed Student's *t* test). Scale bar = 50 μm. Abbreviations: cTNT, cardiac troponin T; DAPI, 4′,6-diamidino-2-phenylindole; EB, embryoid body; FHL2, four-and-a-half-LIM-domain 2.

Differentiating ESC-EBs with stable FHL2 expression appeared to be locked in a mesodermal progenitor state with limited cardiomyocyte differentiation. This block could be reversed by β-catenin/TCF4 inhibition with quercetin. To confirm that transient overexpression of FHL2 during cell commitment would enhance mesodermal cell lineage commitment and subsequently allow for enhanced cardiomyocyte differentiation, we expressed FHL2 transiently in P19 cells (Fig. 7A, 7B; supporting information Fig. S4A). P19 cells were then differentiated in EB cultures in the presence of DMSO (Fig. 7A). Similar to the observations in the stably expressing FHL2-ESCs, qPCR and Western blot analyses showed that FHL2 overexpression enhanced β-catenin transcript abundance (*n* = 6/group, Fig. 7B) and its protein nuclear localization (*n* = 6/group; supporting information Fig. S4B). This also resulted in higher Tcf4 transcript abundance compared to wild-type P19 cells (*n* = 6/group, Fig. 7B). As anticipated, loss in Oct4 expression as well as increased expression of the early cardiogenic transcripts Igfbp5, Tbx5, Nkx2.5 (Fig. 7C), Alpk3, and Mef2a (supporting information Fig. S4C) was observed in FHL2-P19 cells versus control cells at day 2 of differentiation. Augmented expression of Cyclin D1 and of the proliferation marker KI67 was also found in FHL2-P19 cells in comparison to control cells as shown by qPCR and confocal immunofluorescence analysis, respectively (supporting information Fig. S4D, S4E). In contrast to the phenotype exhibited by FHL2-ESCs, transient FHL2 overexpression in P19 cells led to an augmentation of beating EBs in comparison to control P19 cells upon induction of cardiac differentiation (*n* = 12/group; Fig. 7D). Along with this finding, significant loss of mesodermal gene expression (Brachyury, Flk1, and Mesp1; supporting information Fig. S4F) as well as downregulation of the early cardiogenic gene Igfbp5 at 10 days of differentiation was detected (*n* = 6/group, Fig. 7E). cTNT protein and mRNA expression were upregulated in FHL2-transfected cells at 10 days of differentiation (*n* = 6/group, Fig. 7E, 7F). These data suggest that transient FHL2 expression is sufficient to trigger the expansion of cells competent for acquiring a cardiac fate via regulation of β-catenin transcriptional activation.

## DISCUSSION

β-Catenin is the key mediator of canonical Wnt signals, a major regulator of cardiogenesis in vivo and in vitro [7, 28-32]. Tight regulation of Wnt signaling is essential for heart development and cardiac homeostasis [7, 33, 34]. Coactivators and corepressors of Wnt/β-catenin need to be recruited in a tissue- and cell type-specific manner [30, 35]. Identification of cofactors regulating the Wnt canonical pathway may help to increased cardiac differentiation efficiency. Using a Y2H system with β-catenin as a bait, we screened against a human heart cDNA library and identified FHL2 as a novel cardiac β-catenin interaction partner and investigated its role in cardiogenesis in two well-defined cardiogenic cell models (ESCs and P19).

### FHL2 Regulates the Wnt/β-Catenin Pathway in Cardiac Cells

A detailed comparative mRNA expression analysis showed that Fhl2 is expressed in cardiac tissue as early as ED 7.5. This is in line with previous reports showing Fhl2 expression in the earliest myocardial progenitor cells of the embryonic cardiac crescent [36]. Fhl2 expression increased steadily during fetal and postnatal heart development with a peak in the adult heart. Notably, FHL2 protein expression in adult cardiac tissue was particularly high as compared to other organs. Similar observations were reported by Chu et al. [13]. Although these data collectively suggest a role for FHL2 in cardiac development and adult heart homeostasis, genetic deletion of FHL2 did not result in obvious cardiac abnormalities [13]. Importantly, other members of this protein family, such as FHL1 and FHL3, show similar developmental expression patterns as FHL2. However, FHL1 expression was confined to the outflow tract during early development (ED 8.5) and to the cardiac vasculature at later stages [14, 36]. In contrast, FHL3 showed low ubiquitous expression during heart development [14]. Upregulation of FHL1 and FHL3 was not reported in FHL2-deficient mice [13], suggesting little compensatory activity of these related proteins. Notably, FHL1-deficient mice did not exhibit a cardiovascular phenotype [37]; FHL3-deficient mice appear to be not available, yet. We acknowledge that double or even triple knockout models for the three described FHL-family members would be ideal to gain complete insight into their transcriptional role in heart development.

Previous studies showed an interaction between FHL2 and β-catenin resulting in enhanced β-catenin-dependent transcriptional activation in different tumor cell lines as HEK293, SW480, and A375 [9]. In contrast, FHL2/β-catenin interaction resulted in repression of its transcriptional activity in C2C12 myoblasts [8]. In this study, we found FHL2 to interact with β-catenin and to activate β-catenin-dependent transcription in cardiac cells. These data suggest the specific role of FHL2 in Wnt canonical transcriptional regulation in the heart. Biochemical analyses confirmed that nuclear translocation of β-catenin was enhanced upon FHL2 overexpression. Together these findings indicate that FHL2 recruits cell-specific coactivators or coinhibitors to finally control β-catenin-mediated transcription. Our data suggest a prominent cardiac transcriptional role for FHL2, demonstrated by its high expression in the heart and interaction with β-catenin in cardiac cells. Specificity of FHL2 activity in this scenario may be interpreted from data showing only weak interaction of FHL1 and no interaction of FHL3 with β-catenin in HEK293 cells [8] along with a lower expression of FHL1 and FHL3 in the heart [14]. In line with this, we did not find binding between β-catenin and FHL1 or FHL3 in our Y2H screen. Although this does not exclude the possibility of an interaction, it suggests that at least in the heart FHL2/β-catenin is most abundant. Whether this is the case also for diseased myocardium remains to be evaluated.

### FHL2 Promotes Early Cardio-Mesoderm Progenitor Formation During Cardiogenesis

We previously demonstrated that modulation of the Wnt/β-catenin/TCF signaling affects resident cardiac progenitor cell biology [24, 32]. Specifically, using the same Y2H screen, we previously identified and characterized a cardiac Wnt/β-catenin/TCF-dependent transcriptional inhibitor, the Krueppel-like factor (KLF) 15, which was identified as a key-player in resident cardiac progenitor cell fate. In contrast to KLF15, FHL2 enhanced the activation of the Wnt/β-catenin-dependent transcription and was highly expressed in the developing heart, which prompted us to further elucidate the potential of FHL2 in cardiogenesis. ESCs can be cultured as EBs and are able to form the germ layers normally formed during gastrulation. Under appropriate conditions, ESCs have a high propensity for in vitro cardiogenesis and thus represent a robust model system for cardiogenesis [18]. During differentiation, ESCs lose pluripotency, acquire mesodermal characteristics, and subsequently differentiate into cardiac precursors and bona fide cardiomyocytes. Our data demonstrate that stable FHL2 overexpression promotes activation of β-catenin/TCF-dependent transcription and inhibits cardiomyocyte differentiation in a transgenic ESC-EB culture model. Block of cardiac differentiation under FHL2 gain-of-function was attributed to an exacerbated expansion of mesodermal and early cardiac progenitor cells as indicated by increased expression of Brachyury, Flk-1, and Mesp1 as well as Igfbp5, Hand1, Tbx5, and Nkx2.5, respectively. Moreover, FHL2 overexpression resulted in enhanced proliferation and depletion of cells with endodermal characteristics as demonstrated by reduced expression of Hhex and α-Feto-protein. This is in line with previous in vivo data showing that Wnt signaling is essential for the segregation of endodermal and mesodermal populations, in which enhanced Wnt activity directs mesodermal differentiation in the mouse epiblasts [4]. Similarly, signaling gradient can also be formed in ESC-EB cultures [5, 38, 39]. Thus, FHL2 drives ESCs to a mesodermal at the expenses of endodermal differentiation. These findings suggest that FHL2 regulates cell commitment in early layer specification, which is the first step toward cardiac differentiation.

### FHL2-Mediated Wnt/β-Catenin Activation Blocks Terminal Cardiac Differentiation

We showed that FHL2 gain-of-function expands cardiac mesodermal cells by enabling an abnormally persistent expression of genes between undifferentiated mesodermal and early progenitor status. This led to an augmentation of cells with early cardiac identity, but resulted in abrogation of terminal cardiogenic differentiation. Sustained β-catenin/TCF transcriptional activation mediated by FHL2 might explain the described phenotype since inhibition of canonical Wnt signaling is critical for cardiomyocyte differentiation [7, 40]. This conclusion is based on induced inhibition of β-catenin/TCF transcriptional activation in FHL2-ESCs by addition of quercetin, which rescued the cardiac differentiation block induced by FHL2. We previously confirmed that quercetin was able to inhibit β-catenin transcriptional activation in vitro in cardiac progenitor cells [24]. In this study, quercetin-mediated attenuation of FHL2-enhanced β-catenin transcriptional activity resulted in normalized early cardiac gene expression and supported regular cardiomyocyte differentiation. Thus, FHL2 arrested cardiomyocyte differentiation via sustained activation of the β-catenin-dependent transcription.

### Early FHL2-Mediated β-Catenin/TCF Transcriptional Activation Results in Increased Cardio-Mesodermal Specification

Activation of the β-catenin-dependent transcription mediated by FHL2 may induce early cardiac overspecification and thereby block further cardiac differentiation at the cardiomesoderm progenitor stage. This hypothesis was substantiated by the observation of persistent elevated expression of Igfbp5 at 7 and 16 days of differentiation in FHL2-ESC-EBs. Igfbp5 has been identified to mark cells in the precardiac region and early cardiac crescent in mouse embryos [41]. Failure to downregulate Igfbp5 was also observed in Nkx2.5 knockout mice and considered as a sign of progenitor overspecification [41]. We previously reported Igfbp5 upregulation in a mouse model of cardiac-specific β-catenin stabilization [42]. Moreover, isolated cardiac progenitor cells from those mice showed a limited differentiation potential in vitro [32]. We tested the hypothesis that FHL2-mediated overexpression enhances cardiogenesis by directing cells toward the cardiac progenitor stage. Similar to stable expressing-FHL2-ESC-EBs, transient expression of FHL2 in P19-EBs resulted in enhanced early cardiac specification as evidenced by elevated Igfbp5, Nkx2.5, and Tbx5 expression. In contrast to FHL2-ESC-EBs, which maintained significant levels of upregulated Igfbp5 expression at later stages of differentiation, FHL2 transiently overexpressing P19-EBs showed a late significant Igfbp5 downregulation followed by increased cardiomyocyte differentiation. This data support that downregulation of Igfbp5 is necessary for terminal cardiac maturation. Igfbp5 is involved in different cellular processes exerting stimulatory or inhibitory effects depending on its cellular localization [43]. Besides its role as an extracellular growth factor binding protein, Igfbp5 is implicated in direct transcriptional control via its potential for nuclear translocation and DNA interaction [44–46]. Accordingly, we found IGFBP5 in FHL2-ESC-EBs to be predominantly localized in the nucleus at 7 days of differentiation. Activation of another similar member of the Igfbp family, the Igfbp3, was observed in response to activated Wnt3a signaling in a subpopulation of cardiac progenitor cells in vivo [47]. Thus, it is tempting to speculate that Igfbp5 is a downstream transcriptional candidate of the activated Wnt/β-catenin pathway regulating the transition between mesodermal and cardiac mesodermal cells.

### FHL2 Enhances ESC-EBs Proliferation

FHL2 gain-of-function mediated increased cell proliferation in ESC-EBs as indicated by enhanced Cyclin-D1 and KI67 expression. This may subsequently prevent differentiation and further explain the reduced and delayed cardiomyocyte differentiation in ESCs. Whether this process is mediated by β-catenin transcriptional activation, which is known to activate proliferation and the target gene Cyclin-D1 [30, 40], or is a direct effect of FHL2 expression remains to be elucidated. The augmentation in cell proliferation accompanied by the upregulation of the G1/S-specific gene cyclin-D1 is in line with previous reports showing the role of FHL2 in cyclin-D1 activation regulating cell cycle and proliferation [8, 26]. Thus, upregulation of cyclin-D1, mediated directly or indirectly by FHL2 expression, promotes cell proliferation and may further prevent cardiac cell differentiation in ESCs.

Collectively, our observations indicate that FHL2 functions as a cardiac-specific coactivator of Wnt/β-catenin transcriptional activity to control early cell lineage commitment during cardiac differentiation. Previous studies in skeletal muscle cells reported that FHL2 promotes myogenic differentiation via repressing LEF/TCF-dependent transcription [8, 25]. The latter might be explained by recruitment of specific Wnt corepressors by FHL2 in skeletal muscle further suggesting that FHL2 is tuning the Wnt regulation in different cell types.

## CONCLUSIONS

Our findings suggest that FHL2 is an element of the putative cardiogenic differentiation code. In particular, cardio-mesoderm specification induced via Wnt/β-catenin-dependent transcription was enhanced by FHL2. This may in part be mediated by Igfbp5 activation. However, sustained FHL2 overexpression arrested cardiac differentiation at the cardio-mesoderm progenitor stage. This is in agreement with the biphasic role of Wnt/β-catenin in cardiac differentiation. While our study seems to have identified a novel regulator of cardiogenesis in vitro, it has to be acknowledged that *Fhl2*-KO mice do not present obvious defects in cardiogenesis. Whether *Fhl2* deficiency in these mice was compensated by other FHL-protein family members or whether the genetic defect would render them more susceptible to cardiac damage under myocardial stress remains to be studied in more detail. Despite this uncertainty, we provide novel evidence that Wnt/β-catenin modulation with FHL2 may be instrumental in optimization of cardiomyocyte derivation from stem cells for applications in studies of fundamental heart muscle biology, drug discovery, and potentially also cell-based heart repair.

## Supplementary material

Figure S1 Generation of transgenic murine embryonic stem cells overexpressing FHL2. Schematic representation of the analysis of positive clones in transfected double transgenic αMHC-GFP/αMHCNeoR ESCs. Selected clones (8, 7 and 6) were analyzed by southern blot, qPCR and Western blot. (A): Restriction enzymes used for the generation of the antisense riboprobes using the pCS-MT-FHL2 expressing plasmid for Southern blot analysis. (B): Southern blot detection using the riboprobes described in A. Note the presence of multiple copies in the clone 8, which was used in this study as FHL2-overexpressing ESC line. (C): QPCR showing significant up-regulation of Fhl2 transcripts level in the transgenic cell line. Relative mRNA levels were normalized to Gapdh (D): Western blot analysis confirming up-regulation of FHL2 expression in the transgenic line. GAPDH was used as loading control.

Figure S2 FHL2 accelerates reduction in gene expression of undifferentiated cardiogenic cells in ESC-EBs. FHL2 gain-of-function in ESC-EBs resulted in a reduction of the pluripotent gene Oct4, the panmesodermal, and cardiac mesodermal markers Brachyury and Mesp1, respectively, following 7 days of differentiation in comparison to wild-type cells as shown by qPCR. Relative mRNA levels were normalized to Gapdh. Data represent mean ± SEM; n=6, ** p<0.01, *** p<0.001 (two-tailed Student's t-test).

Figure S3 Inhibition of β-catenin-dependent transcription rescues cardiac differentiation in FHL2-ESCEBs. Quercetin (QC) treatment from day 9 of differentiation completely reversed the decrease in αMHC-GFP expression in differentiating FHL2-ESC-EBs (red triangles and discontinuous lines) in comparison to untreated FHL2-ESC-CMs (red dots and continuous lines). αMHC-GFP expression in FHL2-ESC-EBs upon quercetin reaches similar levels of expression as in untreated control ESC-EBs (black dots and continuous lines). αMHC-GFP expression was calculated as percentages of total EBs. Data represent mean ± SEM; n=10, ** p<0.01, *** p<0.001 (two-tailed Student's t-test).

Figure S4 Transient overexpression of FHL2 enhances β-catenin transcriptional activity while promoting cardiogenesis in P19 cells. (A): FHL2 expression in transiently transfected (FHL2), empty vector transfected (CT) as well as undifferentiated (undiff) pluripotent P19 cells shown by Western blot. (B): Nuclear β-catenin accumulation upon FHL2 transient overexpression. Immunoblot of β-catenin in cytosolic and nuclear fractions showing β-catenin nuclear accumulation in FHL2-P19 cells. Semiquantification via densitometric analysis in 3 independent experiments. Normalization was performed with α.tubulin for the cytosolic fraction and Histone H1 for the nuclear fraction. (C): qPCR analysis of an early cardiogenic specification marker Alpk3, and an early cardiogenic marker Mef2a showing increased expression in FHL2-P19 transient-expressing cells at 2 days of differentiation. (D): Up-regulation of the cycling gene Cyclin D1 normalized to Gapdh expression at 2 days of differentiation in FHL2-P19 transiently expressing cells in comparison to CT P19 cells. (E): KI67 (red) expression analyzed by confocal immunofluorescence (percentage of total DAPI positive cells) following 2 days of differentiation demonstrating increased KI67 cell number in FHL2-P19 cells. Representative pictures are depicted. (F): Significantly decreased expression of Brachyury, Flk1 and Mesp1 as determined by qPCR analysis normalized to Gapdh expression at 10 days of differentiation in FHL2-P19 cells. Data represent mean ± SEM; A and B: n=3, C-F: n=6, two-tailed Student's t-test; * p<0.05; ** p<0.01; *** p<0.001. Scale bar: 10 μm.
