# Supplementary figures and images for: The Four and a Half LIM-Domain 2 Controls Early Cardiac Cell Commitment and Expansion Via Regulating β-Catenin-Dependent Transcription
Source: Stem Cells. 2013 Jan 22;31(5):928–40. doi: 10.1002/stem.1332 (PMC3744766; doi:10.1002/stem.1332)

Figure S1

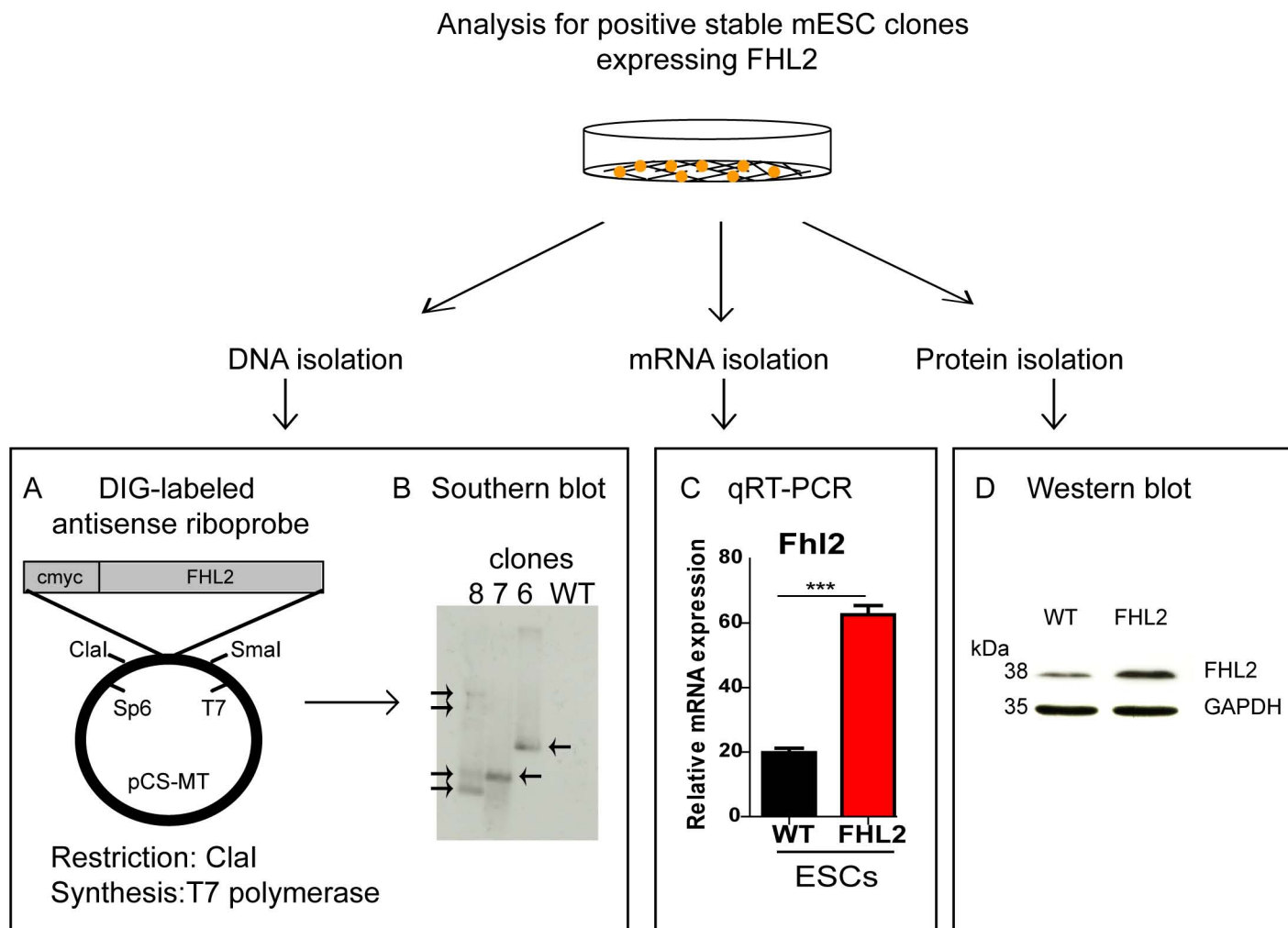

Supplement: Supplementary file 1 [file stem0031-0928-SD1.pdf]

Figure S2

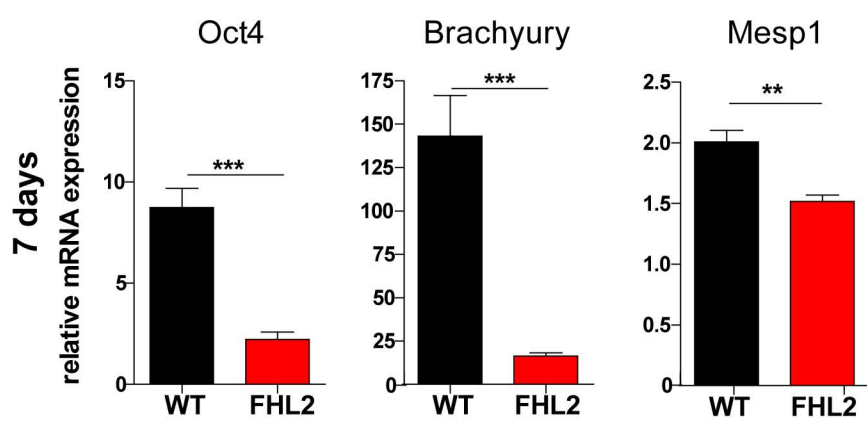

Supplement: Supplementary file 2 [file stem0031-0928-SD2.pdf]

Figure S3

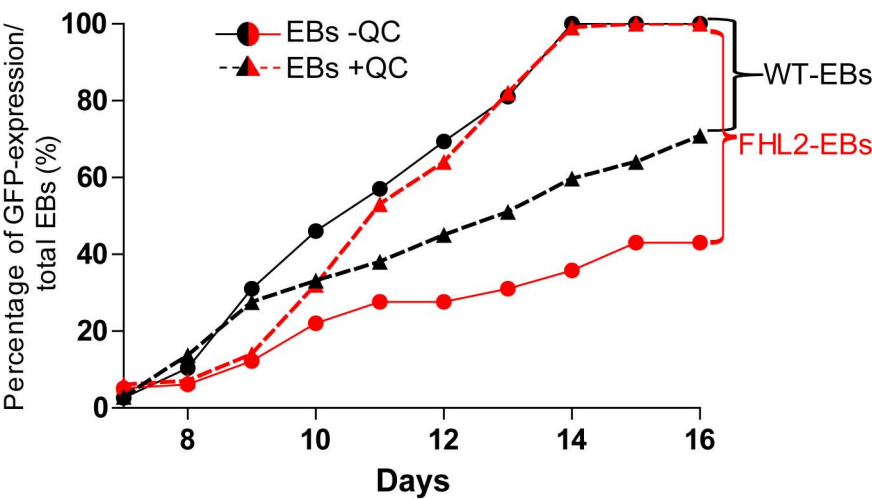

Supplement: Supplementary file 3 [file stem0031-0928-SD3.pdf]

Figure S4

A

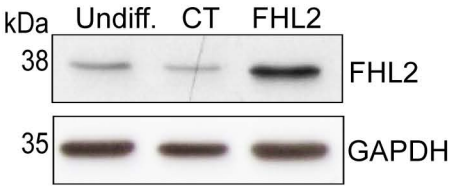

B

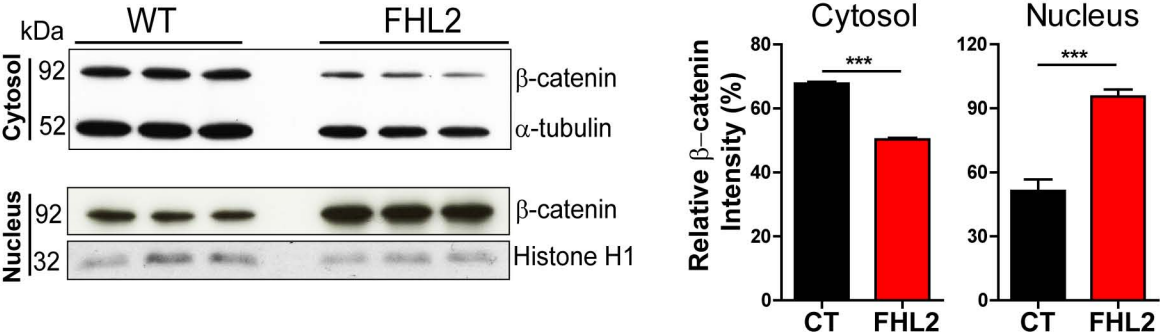

C

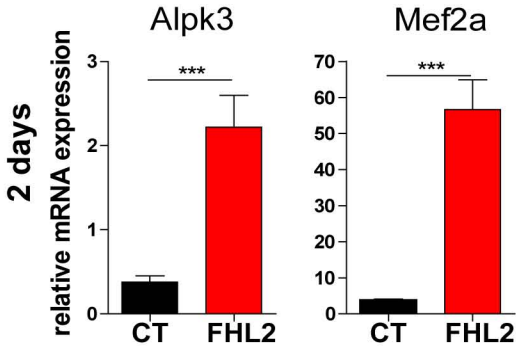

D

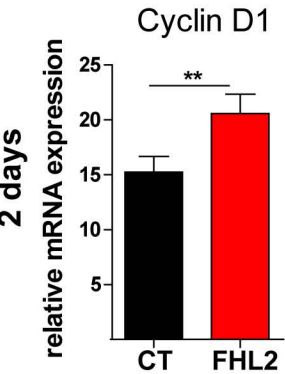

E

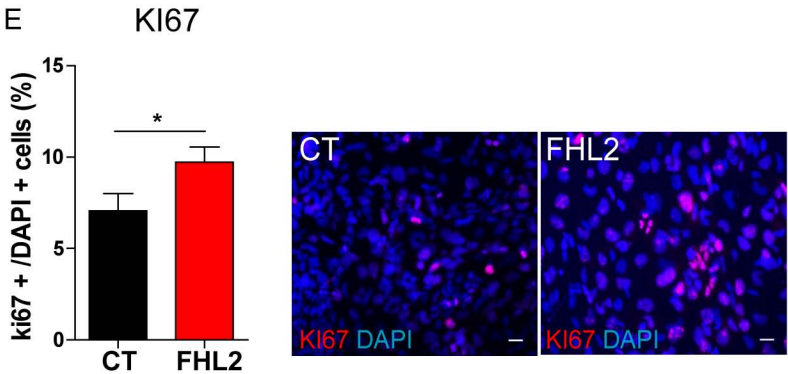

F

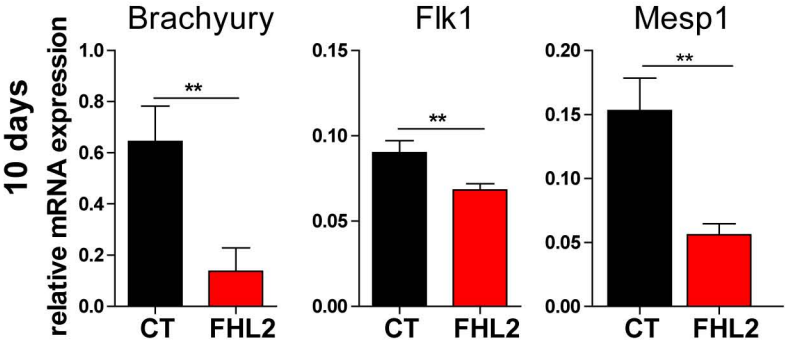

Supplement: Supplementary file 4 [file stem0031-0928-SD4.pdf]
