# Supplementary material for: The Four and a Half LIM-Domain 2 Controls Early Cardiac Cell Commitment and Expansion Via Regulating β-Catenin-Dependent Transcription
Source: Stem Cells. 2013 Jan 22;31(5):928–40. doi: 10.1002/stem.1332 (PMC3744766; doi:10.1002/stem.1332)
Supplement: Supplementary file 5 [file stem0031-0928-SD5.pdf]

**Table S1.** List of primers used in this study.

| Gene | GenBank | Primer | Sequence (5' --> 3') | Fragment length (bp) | Annealing temperature |
|---|---|---|---|---|---|
| β-catenin | NM_007614 | *s* | ACT GCT GGG ACT CTG | 130 | 55°C |
| | | *as* | TGA TGG CGT AGA ACA G | | |
| CyclinD1 | NM_007631 | *s* | CAC CAA TCT CCT CAA CGA C | 149 | 60°C |
| | | *as* | CAC AGA CCT CCA GCA TCC | | |
| Fhl2 | NM_010212 | *s* | GTG CCG ATC CTT GTA GGA CA | 117 | 60°C |
| | | *as* | AAG GAG GAG AAC CCA CAC TG | | |
| Flk1 | NM_010612 | *s* | GGA TGT GGA CTG GGA GGA AG | 286 | 60°C |
| | | *as* | AGA GAT GAG GAA GGA GCA AGC | | |
| Gapdh | NM_001001303 | *s* | ATGTTC CAG TAT GAC TCC ACT CACG | 171 | 60°C |
| | | *as* | GAAGAC ACC AGT AGA CTC CAC GACA | | |
| Gata4 | NM_008092 | *s* | CTG GAG GCG AGA TGG | 157 | 60°C |
| | | *as* | GGT GGT GGT AGT CTG G | | |
| Hand1 | NM_008213 | *s* | GTG GCA AGT CCG CAG AAG | 291 | 60°C |
| | | *as* | GGT GTG AGT GGT GAT GAT GG | | |
| Igfbp5 | NM_010518 | *s* | AGG TGG TGA CAG AGC AGG TG | 130 | 60°C |
| | | *as* | GCC GCA GAA CAG GTA AGA GG | | |
| Mef2a | NM_001033713 | *s* | ACA CCA ACC AGA ACA TC | 203 | 60°C |
| | | *as* | GTC ACT GCC ATC ATA GG | | |
| Mesp1 | NM_008588 | *s* | GGT AGC GGA CAG CGG CAG AG | 102 | 60°C |
| | | *as* | CAC GGA TGG CGG CAA GAA GC | | |
| Nkx2.5 | NM_008700 | *s* | CGA CAG CGG CAG GAC CAG AC | 133 | 60°C |
| | | *as* | CGT AGG CGG GAG CGT AGG C | | |
| Oct4 | NM_013633 | *s* | GCT TCA GAC TTC GCC TCC TCA CC | 191 | 60°C |
| | | *as* | GCC ATC CCT CCG CAG AAC TCG | | |
| Tbx5 | NM_011537 | *s* | GCA AGC AAT CCC CAG CAC AAA C | 183 | 60°C |
| | | *as* | GCC AAA GCC CTC ATC TGT ATC GG | | |
| Tcf4 | NM_001083967 | *s* | AAC GGA ACA GAC AGT ATA ATG G | 147 | 60°C |
| | | *as* | CAC AGG AGT TGA AGG ATT GG | | |
| Tnnt2 (cTnT) | NM_011619 | *s* | AAG CAG CAG AAA TAC GAA ATC AAC ( | 183 | 60°C |
| | | *as* | GCC AAG GAG GAC CCA GAG C | | |
| MLC2A | NM_022879 | *s* | TCA GCT GCA TTG ACC AGA AC | 148 | 60 °C |
| | | *as* | AAG ACG GTG AAG TTG ATG GG | | |
| NCAM | NM_010875 | *s* | GTC TGT CAC CCT GGT GTG TG | 63 | 60 °C |
| | | *as* | ATC CTT TGT CCA GCT CAT GG | | |
| Brachuyry T | NM_009309 | *s* | CCG GTG CTG AAG GTA AAT GT | 274 | 60 °C |
| | | *as* | CCT CCA TTG AGC TTG TTG GT | | |
| Hhex | NM_008245 | *s* | ACG ACT ACA CGC ACG CCC TAC | 110 | 60°C |
| | | *as* | GAA CCT CAC TTG ACC GCC TTT CC | | |
| α-Fetoprotein | NM_007423 | *s* | CTTGGTGAAGCAAAAGCCTGAA | 123 | 60°C |
| | | *as* | GGACCCTCTTCTGTGAAACAGACT | | |
